# Interventions of the clinical pharmacist in an Intermediate Care Unit for elderly patients

**DOI:** 10.1590/S1679-45082017AO3894

**Published:** 2017

**Authors:** Stéphanie de Souza Costa Viana, Tiago Arantes, Sabrina Corrêa da Costa Ribeiro

**Affiliations:** 1 Divisão de Farmácia, Instituto Central, Hospital das Clínicas, Faculdade de Medicina, Universidade de São Paulo, São Paulo, SP, Brazil.; 2 Departamento de Emergências Clínicas, Hospital das Clínicas, Faculdade de Medicina, Universidade de São Paulo, São Paulo, SP, Brazil.

**Keywords:** Health of the elderly, Pharmacists, Drug therapy, Critical care, Pharmacy service, hospital, Saúde do idoso, Farmacêuticos, Tratamento farmacológico, Cuidados críticos, Serviço de farmácia hospitalar

## Abstract

**Objective:**

To discuss the role of the clinical pharmacist in hospital care of critical elderly patients.

**Methods:**

Critical patients aged 60 years and over admitted by the clinical staff to an Intermediate Care Unit were followed-up for 4 months regarding their drug therapies. Medical prescriptions were reviewed daily on the basis of patients’ clinical conditions, with the view to identify opportunities for optimization of drug therapies, contributing to safer prescribing, reduced discomfort and correct and rational use of drugs.

**Results:**

A total of 386 prescriptions were reviewed and 212 pharmaceutical interventions performed; 64.3% of prescriptions were classified as accepted with changes, 28.5% not accepted and 7.2% verbally accepted with no changes. Interventions included drug therapy indications, directions for dose adjustment, reduction of the use of potentially inappropriate medications for older patients, prescription adjustments, discontinuing unnecessary drugs, among others.

**Conclusion:**

The significant number of interventions accepted by the healthcare staff supports the relevance of the clinical pharmacist as a member of the multiprofessional team, especially in care of the elderly.

## RESUMO

**Objetivo:**

Discutir o papel do farmacêutico clínico no cuidado hospitalar de pacientes críticos, idosos.

**Métodos:**

Ao longo do período de 4 meses, foi realizado o acompanhamento farmacoterapêutico de pacientes críticos com 60 anos ou mais, admitidos pela equipe de Clínica Médica em uma Unidade de Cuidados Intermediários. Diariamente, foram realizadas avaliações das prescrições médicas, frente ao quadro clínico, a fim de encontrar oportunidades de otimização da farmacoterapia prescrita, contribuindo para a maior segurança da prescrição, a redução de desconfortos, e o uso correto e racional de medicamentos.

**Resultados:**

Foram avaliadas 386 prescrições e realizadas 212 intervenções farmacêuticas, sendo 64,3% destas classificadas como aceitas com alteração na prescrição, 28,5% não aceitas e 7,2% aceitas verbalmente, porém sem alteração na prescrição. As intervenções envolviam indicações farmacoterapêuticas, orientações para ajustes de dose, redução do uso de medicamentos potencialmente inapropriados para idosos, ajustes de prescrição, suspensão de medicamentos desnecessários, entre outras.

**Conclusão:**

O significativo número de intervenções aceitas pela equipe da saúde reforça o papel que o farmacêutico clínico tem a desempenhar na equipe multiprofissional, sobretudo quando direcionados ao cuidado de idosos.

## INTRODUCTION

The epidemiological transition is a concept that refers to the long-term modification of morbidity, mortality and disability patterns that characterize a specific population, and generally coincide with other demographic, social, and economic transformations. It is estimated that, in 2025, Brazil will have the sixth largest population of older people in the world, with approximately 35 million individuals aged 60 and over. This fact poses increasing challenges to health care services, due to the special care required for this age range.^(^ ^1^ ^,^ ^2^ ^)^

Increased life expectancy may result in people living with chronic noncommunicable diseases (CNCD) for a much longer time, with the need for ongoing medical follow-up and polypharmacy. We know that the reduced reserve capacity and reaction to injury of the elderly can more easily leave them in a critical state requiring intensive care in specialized health clinics.^(^ ^2^ ^-^ ^5^ ^)^

Aging involves physiological changes that can lead to pharmacokinetic (such as increased half-life and serum concentrations of drugs) and pharmacodynamic changes, supporting the need for drug-therapy monitoring and dose adjustment, especially at this age range. The possibility of drug-induced damage, even when drugs are used at recommended doses and for the correct indication, is a major problem for elderly inpatients.^(^ ^5^ ^-^ ^8^ ^)^

The incidence of adverse events in Intensive Care Units (ICU) is 19 events per 1,000 patients per day, compared to 10 events per 1,000 patients per day in other care settings.^(^ ^9^ ^)^

The term ‘clinical pharmacy’ is used to describe the actions performed by the pharmacist in favor of the patient, *i.e.* identification, resolution, and prevention of drug-related problems (DRP). These actions include reviews of hospital drugs and medical prescriptions to reduce the associated risks, which contributes to better disease management and shorter length of stay, reduced DRP and mortality. Moreover, it leads to financial benefits and ensures the safety and effectiveness of the therapy prescribed, as well as its rational use by multidisciplinary teams at different care levels.^(^ ^4^ ^,^ ^7^ ^,^ ^10^ ^-^ ^13^ ^)^

Polypharmacy includes at least one drug unnecessary to the patient’s drug therapy,^(^ ^1^ ^)^ raising the risk of drug-related toxicity when comorbidities are present.^(^ ^5^ ^)^ In addition, the use of potentially inappropriate medication (PIM) for older people, as defined by the Beers criteria, may cause confusion, cognitive impairment, worsening of clinical symptoms and increased mortality.^(^ ^14^ ^)^

Multiprofessional approaches create relations that facilitate the exchange of knowledge and skills, contributing to broader and better patient care, and offering benefits to patients, particularly the elderly.^(^ ^13^ ^)^ Evidence shows that collaborative work between pharmacists and physicians improves patient care, and that teamwork is critical to the safety and efficacy of care provided.^(^ ^10^ ^)^

## OBJECTIVE

To discuss the role of the clinical pharmacist in hospital care of critical, elderly patients.

## METHODS

A prospective and descriptive study, with data collection from October 2015 to January 2016, conducted at the Intermediate Care Unit of *Hospital das Clínicas da Faculdade de Medicina da Universidade de São Paulo*. The project was approved by the Ethics Committee of the organization under opinion number 1.266.550 and CAAE: 49225015.5.0000.0068, and authorized by the unit management. The requirement for an Informed Consent Form was waived by the Committee.

The Intermediate Care Unit, also known as the Critical Emergency Unit, only admits patients coming from the Emergency Department with clinical conditions acute or chronic decompensated that life-threatening, requiring artificial support interventions, such as mechanical ventilation and the use of vasoactive drugs, as well as intensive monitoring for any intervention. The unit has 17 beds, 12 of which are for clinical and 5 for surgical patients, used for monitoring critical patients in emergency situations when the ward is full and there are no beds available in the conventional ICU.^(^ ^15^ ^)^

The activities in this study aim to ensure continuity of the weekly service provided by pharmacists during multiprofessional visits to older patients.

Patients aged 60 years or older admitted for at least 24 hours into this hospital unit and seen by the Internal Medicine team were enrolled in this study. We excluded patients who stayed for less than 24 hours, as well as patients admitted or discharged during weekends and holidays.

We used the hospital computer system to identify the admissions within the period and access patient data, as well as data of the unit the patient had been assigned. This system provided the charts and medical prescriptions, allowing for daily monitoring of the patient, in order to optimize the drug therapy prescribed. This information was complemented by a review of nursing controls (blood pressure, glycemia, temperature, and presence of bowel movements) and the paper prescription at the unit (due to manual changes). The suitability of the prescribed drugs was assessed in view of the most frequent needs of these patients, such as analgesia, venous thromboembolism (VTE) prophylaxis, stress ulcer prophylaxis, use of laxatives, antimicrobials and laboratory tests. Drug interactions were assessed via Micromedex^®^ and UptoDate^®^. To identify and assess the risks associated with the use of PIM, we consulted the second table in the Beers criteria, which lists the drugs that should be avoided for many or most elderly patients, except those on palliative care or in hospices*.* ^(^ ^14^ ^)^

Prescriptions were reviewed for the need for the prescribed medication; presentation and suitability to the clinical condition; dose, taking into account renal function, age, the serum level of drugs and liver function; scheduling by the nursing staff; need to add new items to the drug therapy due to clinical status.

The interventions were carried out in person with the medical and nursing staff, in order to define the best management to be followed. We also assessed the use of inhalation drug delivery devices and guidance provided.

This information was recorded in a standard form and a Microsoft Excel^®^ worksheet, and the outcomes were monitored on subsequent days.

Acceptance of interventions was divided into accepted with changes in the prescription; verbally accepted with no changes in the prescription, and not accepted.

## RESULTS

**Figure 1:**
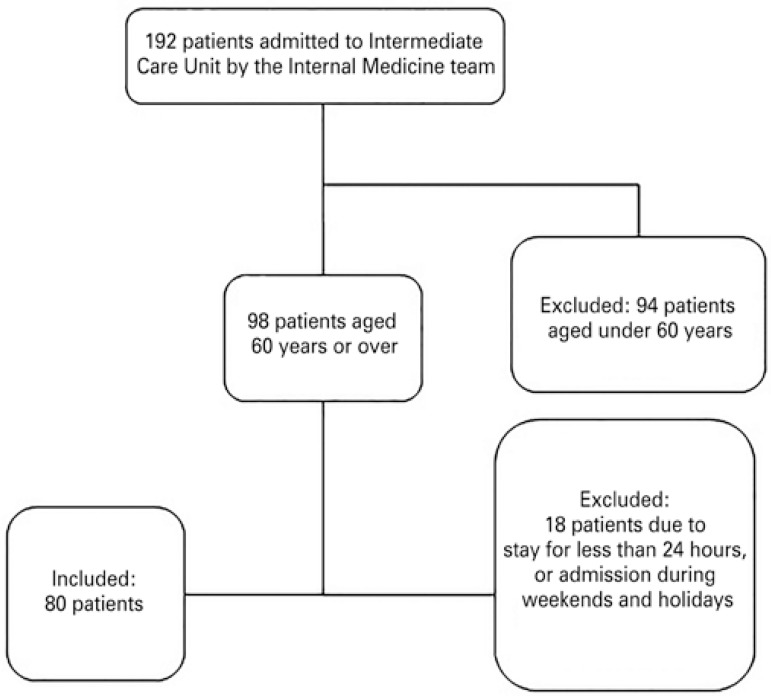
Patient enrollment flow chart

According to the inclusion criteria, 81.6% of the total number of elderly patients admitted by the Internal Medicine team were monitored over the course of the study. At the time, 51% of patients assigned to this team at the unit were aged 60 years or older (Figure 1).

The sample consisted of 53.7% women (Table 1), the mean age of 72.5 years, and the highest recorded age was 94.

**Table 1:** Characteristics of elderly patients monitored by pharmacists over the course of the study

Patient characteristics (n=80)	n (%)
Age group, years	
60-69	38 (47.5)
70-79	25 (31.2)
80 or older	17 (21.3)
Sex	
Male	37 (46.3)
Female	43 (53.7)
Main causes of hospitalization*	
Circulatory system diseases	33 (41.3)
Respiratory system diseases	14 (17.5)
Certain infectious and parasitic diseases	13 (16.3)
Main comorbidities	
Systemic arterial hypertension	58 (72.5)
*Diabetes mellitus*	34 (42.5)
Dyslipidemia	17 (21.2)
Previous stroke	10 (12.5)

* The main causes of hospitalization were classified according to the 10^th^ revision of the International Statistical Classification of Diseases and Related Health Problems of the World Health Organization, updated in 2016.^(^ ^16^ ^)^

In addition to the main cause of hospitalization, other comorbidities were identified, such as systemic arterial hypertension (72.5%), type 2 *diabetes mellitus* (43%), liver disease (8.8%) and renal disease (6.2%), for example, raising the complexity of patient care and therapy regimens.^(^ ^8^ ^)^

The mean length of stay in the Intermediate Care Unit was 10 days, with 46.3% resulting in discharge home, 46.2% death and 7.5% transfer to other buildings in the complex.

As for the drug therapy, the average number of drugs used daily was 12 (minimum 5 and maximum 24 items per prescription) among 386 prescriptions reviewed. A total of 212 pharmaceutical interventions were performed in 62 patients (77.5%), with an average of 3 interventions per patient, classified as therapy indications and interventions for the rational use of drugs.

Therapy indications corresponded to 38.7% of interventions, *i.e.*, the patient needed a certain drug that had not been prescribed (Table 2).

**Table 2:** Drug therapy indications to the medical staff

Therapy indications performed	n (%)
Laxatives	40 (48.8)
Other drugs (correction of hydroelectrolytic imbalances, absence of required medications, among others)	21 (25.6)
Stress ulcer prophylaxis	8 (9.8)
Venous thromboembolism prophylaxis	5 (6.1)
Analgesia	5 (6.1)
Drug therapy alternatives	3 (3.6)

In 48.8% of these interventions, the purpose was to adopt laxative measures for the patient in the absence of bowel movements for at least 3 days, according to nursing controls. The indication of measures to correct hydroelectrolytic imbalances and other drugs required for the patient’s clinical condition, such as inhalation drug delivery devices and measures to control sialorrhea, for example, occurred in 25.6%.

Prophylaxis of stress ulcer and VTE was absent in 9.8% and 6.1% of prescriptions, respectively, when there was an indication and this resulted in new interventions. The indication of analgesic agents occurred in 6.1% of cases, aiming to reduce discomfort caused by pain and worsening of the quality of life throughout hospitalization.

The other pharmaceutical indications were suggestions of more appropriate therapeutic alternatives, favoring the effective use of the drug.

Approximately 14% of interventions were due to medications prescribed without an indication for the patient’s clinical condition. Other 14.1% of interventions were related to the need of adjusting prescriptions due to differences between the chart and the prescription, and even the absence of elements that were required for prescription, such as the dosage. There was a need for dose adjustment due to renal function in 11.3% of cases.

PIMs were identified 196 times in the prescriptions reviewed, but they were recorded only once, when they first appeared, with special note to metoclopramide, haloperidol, amiodarone, and quetiapine, corresponding to 7.5% of the interventions performed.

Dose adjustments were suggested in 3.8% of interventions in order to ensure the effectiveness of the drug, and adequacy of antimicrobials, based on sensitivity testing and for the right duration, in 1.9% (Table 3).

**Table 3:** Pharmaceutical interventions performed with the medical staff

Pharmaceutical interventions performed	n (%)
Use of medication without indication	30 (14.1)
Need for adjustments in prescription	30 (14.1)
Need for dose adjustment due to renal function	24 (11.3)
Use of potentially inappropriate medication for older patients	16 (7.5)
Need for dose adjustment	8 (3.8)
Inappropriate use of antimicrobials	4 (1.9)

To ensure the best use and efficacy of inhalation drug delivery devices used by patients with respiratory tract diseases, we provided guidance on the correct use to five patients who had been using them in the wrong way. In these cases, interventions were not classified in terms of acceptance.

Changes in prescriptions or requests for laboratory tests due to drug interactions accounted for 2.4% and, in the majority of the cases, the drug use was monitored. These cases were not recorded.

To prevent intoxication and lack of effectiveness of the drug, laboratory tests were requested to assess serum levels of the drugs (such as phenytoin and vancomycin) and electrolytes in 3.8% of all interventions, allowing for adjustments as needed.

**Figure 2:**
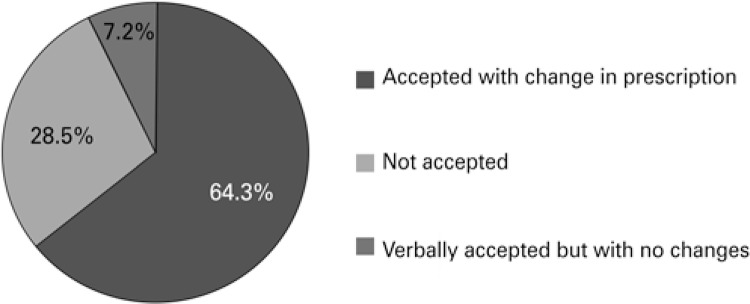
Acceptance of interventions

A total of 64.3% of interventions were accepted with changes in the prescription, 28.5% were not accepted, and 7.2% were verbally accepted with no changes in the prescription (Figure 2).

The interventions performed with the medical staff accounted for 97.6% of total, and those performed with the patients and the nursing staff, 2.4%; of these, 46% with the purpose of ensuring patient safety by preventing associated risks; 24% to improve comfort by reducing avoidable symptoms; 12.7% to reduce costs by promoting the rational use of drugs; 11.7% to improve the effectiveness of drug use; and 5.6% to prevent prescription-associated errors.

## DISCUSSION

The main causes of hospitalization identified in this study reflect the CNCDs presented by a large number of elderly patients. According to studies, systemic arterial hypertension is accountable for the high morbidity and mortality rates of this age group^(^ ^1^ ^)^ and *diabetes mellitus* is the sixth leading cause of hospital admissions in Brazil, significantly contributing to other causes, such as stroke.^(^ ^17^ ^)^ The concomitant presence of these diseases increases the mortality and disability rates due to the harm they cause.^(^ ^13^ ^)^

As for hospitalization outcomes, 46.2% of patients died. This mortality rate was lower than the estimated 56.8%, based on the Simplified Acute Physiology Score (SAPS) III, adjusted for Latin America.

During the pharmaceutical monitoring period, there was an indication for laxatives in 48.8% of cases. Constipation in critically ill patients is often related to some factors, such as diet, drug use, dehydration, bed rest, among others, and may lead to worse clinical outcomes. Therefore, it should be actively managed to reduce the length of stay, the incidence of *delirium*, duration of mechanical ventilation, the length of ICU stay, as well as to increase the quality of life.^(^ ^18^ ^,^ ^19^ ^)^

Gastric bleeding triggered by stress ulcers is one of the most frequent complications of ICU patients due to the physiological, inflammatory and hemodynamic stress to which they are submitted.^(^ ^20^ ^)^

Some factors, such as restricted mobility, previous comorbidities, advanced age, among others, are related to a higher risk of VTE in the elderly, and the type and duration of immobilization must be taken into account.^(^ ^21^ ^)^

The interventions related to the inclusion of prophylaxis for these complications (9.8% and 6.1%) aim to reduce morbidity and mortality rates and the risk of a longer stay at the unit.

The use of drugs with no indication increases the risk for drug interactions, the rate of adverse reactions and avoidable costs, besides affecting the patient health status. In our study, 66.7% of these interventions were classified as accepted, and 10% as verbally accepted with no changes – out of 30 interventions performed.

Due to the changes in renal function associated with aging, the use of tools that estimate the glomerular filtration rate to allow for dose adjustments is essential. In our study (11.3%), we used the Cockroft-Gault and the Modification of Diet in Renal Disease (MDRD) equations, at the discretion of the medical team, despite the differences shown in previous studies between these methods,^(^ ^22^ ^)^ supporting the importance of standardization.

The interventions to discuss the use of PIM accounted for 7.5% of the total suggestions made. Except for amiodarone, the other drugs (haloperidol, quetiapine, and metoclopramide) were mostly prescribed *pro re nata* (p.r.n.), and when regular use was detected, pharmaceutical interventions were performed suggesting the use of a safer alternative, if possible. Antipsychotic drugs are associated with increased risk of stroke, cognitive decline, and mortality in patients with dementia; metoclopramide was associated with increased risk of extrapyramidal symptoms and tardive dyskinesia, particularly in frail, elderly patients,^(^ ^14^ ^)^ and should, therefore, be avoided. Other drugs, such as amitriptyline, carisoprodol, cyclobenzaprine, were kept based on their risk-benefit ratio as assessed by the medical staff, despite the possibility of anticholinergic effects, for example.^(^ ^14^ ^)^ The use of these drugs was monitored in the following days, and they ranged in number from one to five in the applicable prescriptions.

Clinical pharmacists are very important in patient care since they ensure the safe and rational use of medications. Drug therapy monitoring decreases medication errors by 78%,^(^ ^23^ ^)^ and improves the quality of prescriptions, reducing the incidence of adverse events.^(^ ^13^ ^)^

The acceptability of interventions in our study (71.5%, 64.3% of which with changes) was similar to that observed in another study with similar methodology at a university hospital, in the State of Paraná (76.3%).^(^ ^1^ ^1^ ^)^

The rate of not accepted interventions reflects the need for improvement, better approximation of clinical experience, as well as setting the right time for concise and more in-depth interventions, contributing more significantly to the results. The instability of critically ill patients resulted, in part, from the verbally accepted interventions with no change to the prescriptions, due to the rapid progression to death in some cases. The other fractions reflect transfers to other clinics and other factors that we could not identify in this study.

## CONCLUSION

The inclusion of clinical pharmacists in the healthcare team allows for better monitoring of patients’ clinical condition through rational and safer prescriptions, and it also contributes to care provided by the medical staff. The importance of this professional, especially in the care of critical elderly patients, lies in the monitoring and control of the use of low-therapeutic-index drugs and potentially inappropriate medications; follow-up and providing recommendations for dose adjustment in view of renal function; and the proper use of drugs, contributing to reducing discomfort and achieving full recovery. The significant number of interventions accepted by the healthcare team supports the relevance of the clinical pharmacist in the multiprofessional team, especially in care of the elderly.
